# A novel *CUL4B* splice site variant in a young male exhibiting less pronounced features

**DOI:** 10.1038/s41439-019-0074-6

**Published:** 2019-09-04

**Authors:** Yuji Nakamura, Yusuke Okuno, Hideki Muramatsu, Tomoko Kawai, Kazuhito Satou, Daisuke Ieda, Ikumi Hori, Kei Ohashi, Yutaka Negishi, Ayako Hattori, Yoshiyuki Takahashi, Seiji Kojima, Shinji Saitoh

**Affiliations:** 1 Department of Pediatrics and Neonatology, Nagoya City University Graduate School of Medical Sciences, Nagoya, Japan; 2 Center for Advanced Medicine and Clinical Research, Nagoya University Hospital, Nagoya, Japan; 3 Department of Pediatrics, Nagoya University Graduate School of Medicine, Nagoya, Japan; 4 Department of Maternal-Fetal Biology, National Research Institute for Child Health and Development, Tokyo, Japan; 5 Department of Genome Medicine, National Research Institute for Child Health and Development, Tokyo, Japan

**Keywords:** Genetics research, Outcomes research

## Abstract

Patients with variants in *CUL4B* exhibit syndromic intellectual disability (MIM #300354). A seven-year-old boy presented with intellectual disability, a history of seizure, characteristic facial features, and short stature. Whole-exome sequencing detected a c.974+3A>G variant in *CUL4B*, which was subsequently confirmed to disrupt mRNA splicing. The current patient showed less pronounced phenotypic features compared with the previously reported cases. This report, therefore, provides evidence of genotype–phenotype correlations in *CUL4B*-related disorders.

*Cullin 4B* (*CUL4B*), containing 22 exons located in chromosome Xq24, is a member of the cullin-RING ubiquitin ligase family that controls a wide spectrum of cellular processes^1^. Tarpey PS et al. first revealed that mutations in *CUL4B* cause the Cabezas type of X-linked syndromic intellectual disability (ID)^2^. Since then, at least 83 cases from 56 families with variants in *CUL4B* have been reported, and are currently estimated to account for about 3% of the X-linked ID population^3–10^. Whole-exome sequencing (WES) has identified a number of pathogenic variants in patients with *CUL4B*-related ID. However, intronic candidate variants, particularly those outside canonical ±1 or 2 splice sites, are difficult to assess and therefore might be unreported. Furthermore, despite *CUL4B* variants being reported in at least seven cases under 10 years of age^8,9,11,12^, the clinical features in early childhood are yet to be fully elucidated. Herein, we report another case of a patient with a variant at intronic +3 position in *CUL4B*.

The patient was a seven-year-old boy who had a regular follow-up in our hospital due to developmental delay. He was the first of two children from healthy non-consanguineous Japanese parents. No family members, including his younger sister, had any neurological disease. He was born after 38 weeks of an uneventful pregnancy via cesarean section due to non-reassuring fetal status, with Apgar scores of six and nine at one and 5 min after birth, respectively. His birth weight was 2164 g (−2.0 SD), length was 44.5 cm (−1.8 SD), and head circumference was 34.0 cm (+0.7 SD). His neonatal period was uneventful, but it was noted that he was easily excited since infancy. Developmental delay became evident with sitting not observed until 13 months, and unassisted walking and speaking of some meaningful words not until three years and four months of age. Autistic behaviors became more apparent as he became older. A febrile seizure occurred once, at 2 years of age. A brain magnetic resonance imaging (MRI) at four years of age showed only persistent cavum septum pellucidum (Fig. 1a). Laboratory diagnostic workup was normal, and his karyotype, determined by chromosomal G-band testing, was 46,XY. At present, his weight is 14.7 kg (−2.1 SD), length is 105.3 cm (−3.3 SD), and head circumference is 52.8 cm (+0.4 SD), indicating short stature and relative macrocephaly. He showed sandal gap and distinctive facial features, namely prognathia, protruding and cupped ears, and low nasal bridge. A neurological examination revealed no motor or sensory deficits. He spoke two-word sentences, and received special education services.

**Fig. 1:**
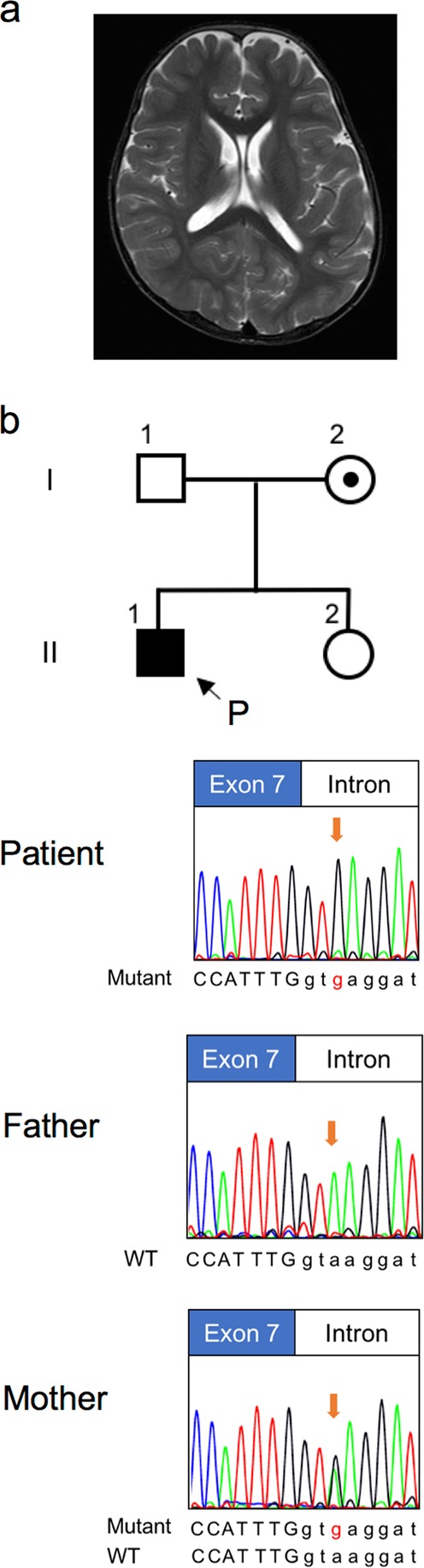
Clinical features, familial pedigree, and Sanger sequencing of the variant. **a** Brain MRI (T2 weighted image) of the patient at the age of four years showed cavum septum pellucidum. **b** Familial pedigree of the *CUL4B* variant and identified variants in genomic DNA. The arrow denotes the proband. Note the single peak of guanine (black line) in the patient representing a hemizygous sequence variant of c.974+3A>G

After obtaining written informed consent for genetic studies, we performed trio-based WES analysis using the SureSelect Human All Exon V6 kit (Agilent Technology, Santa Clara, CA, USA) for capture and a HiSeq2500 (Illumina, San Diego, CA, USA) for sequencing with 101-bp paired-end reads. To identify a disease-causing variant, we excluded variants with a minor allele frequency >1% in public databases. The public databases used were 1000 Genomes Project; Exome Aggregation Consortium; National Heart, Lung, and Blood Institute Exome Sequencing Project 6500; and Human Genetic Variation Database. After the filtering process, we identified a c.974+3A>G variant in *CUL4B* (NM_003588.3) in the patient and his mother, which was subsequently validated by Sanger sequencing (Fig. 1b). The novelty of this newly identified variant was confirmed by querying The Human Gene Mutation Database® (professional release 2019.2) and National Center for Biotechnology Information ClinVar database. In silico analysis using Alamut® Visual version 2.1, which included five prediction algorithms (SpliceSiteFinder-like (SSF-like), MaxEntScan (MES), NNSPLICE, GeneSplicer, and Human Splicing Finder), calculated a moderate decrease in scores, indicating a possible splicing effect (Fig. 2a). To confirm the effect of the c.974+3A>G variant, which is located in splice-donor site 3 base pairs downstream of exon 7, RT-PCR with primers binding to exons 5 and 11 of *CUL4B* was performed using total RNA from peripheral leukocytes. Agarose gel electrophoresis of the PCR-amplified cDNA products revealed this variant, located at an exon-intron boundary, resulted in two different aberrant splicing transcripts of *CUL4B* (Fig. 2b). Sequence analysis of the cDNA products, which were extracted from the electrophoresed gel, revealed these aberrant transcripts lack either exon 7 or both exons 7 and 8 (Fig. 2c). The transcript lacking exon 7 alone is assumed to result in a frame-shift leading to a premature stop codon (p.Ile283Glyfs*8). The transcript lacking both exon 7 and 8 is assumed to result in an in-frame major length change (p.Met284_Ile362del), which disrupts the cullin domain located between amino acid residues 217 and 815. Thus, this indicates c.974+3A>G is a pathogenic variant.

**Fig. 2:**
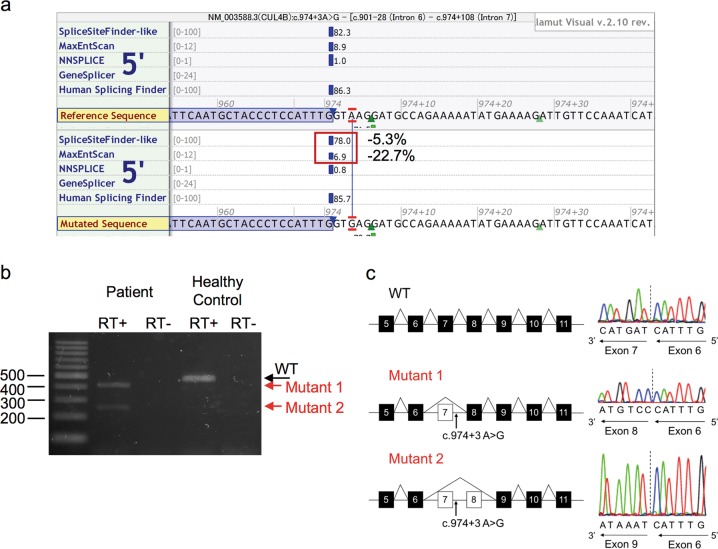
Analysis of the splice site variant. **a** Alamut® splicing window with differences highlighted. The scores next to vertical blue bars indicate predicted 5’ splice sites. Note the 5.3% decrease in SSF-like, and 22.7% decrease in MES, with a potential effect on splicing. **b** RT-PCR analysis in the patient and a healthy control. Two products are amplified in the patient (lane 2, indicated by the red arrows, labeled ‘Mutant 1’ and ‘Mutant 2’), whereas only a single product is amplified in the control (lane 4, indicated by the black arrow). **c** Schematic representations of sequenced cDNA products. The entirety of exon 7 (74 bp) and both exons 7 and 8 (total 237 bp) are skipped in Mutant 1 and 2, respectively. Electropherograms represent antisense strands

In this report, we identified a novel *CUL4B* splice site variant at c.974+3 in a patient with syndromic ID, with in silico tools predicting possible pathogenicity in the A>G variant. The prediction was considered deleterious because it met Houdayer’s criteria, including a decrease of more than 5% and 15% from wild type scores using SSF-like and MES, respectively^13^. The intronic variant was experimentally confirmed by RT-PCR to disrupt the splice-donor site. Our data indicate that an A>G substitution in the intronic +3 position may be an important pathogenicity mechanism. We also show how in silico methods provide a potential tool for initial assessment of intronic candidates, especially outside canonical ±1 or 2 splice sites.

Previous reports have described phenotypic features caused by mutations in *CUL4B*. Compared with the 83 previously reported cases, the phenotypic features of our patient were less pronounced, lacking some of the common features, such as obesity, tremor, and hypogonadism^3–10^. To account for the reduced phenotypic expression observed in our patient, we first searched for genotype–phenotype correlations, but no significant trends were identified. We then stratified the clinical features of our patient and the reported patients by age, where information was available (Table 1). This analysis revealed that the rate of obesity, tremor, gynecomastia, and hypogonadism was much lower in affected individuals under 10 years of age compared with affected individuals over 10 years of age^3,8,11,12,14,15^. Thus, the reduced phenotypic expression observed in the current patient might be explained by age-dependent penetrance.

**Table 1:** Phenotypic features of the current patient and previously reported patients under 10 years and over 10 years of age with mutations in *CUL4B*

	Current case	<10 years (ref.^8,9,11,12^)	≥10 years (ref.^3,8,11,12,14,15^)
Number of patients	1	7	22
Median age, year	7	5 (2–8)	28 (10–41)
Growth			
Short stature	+	2/7 (29%)	21/22 (95%)
Obesity	−	1/6 (17%)	10/16 (63%)
Neurological			
Intellectual disability	+	7/7 (100%)	21/22 (95%)
Motor delay	+	4/4 (100%)	17/18 (94%)
Speech impairment	+	6/7 (86%)	23/24 (96%)
Behavioral problems	+	5/7 (71%)	15/22 (68%)
Tremor	−	0/4 (0%)	9/15 (60%)
CNS abnormality	+	4/4 (100%)	7/9 (78%)
Seizure	+	3/3 (100%)	2/5 (40%)
Craniofacial			
Macrocephaly	+	5/7 (71%)	5/21 (24%)
Malformed/abnormally positioned ears	+	5/6 (83%)	11/12 (92%)
Narrow palpebral fissures	−	4/6 (67%)	11/14 (79%)
Low nasal bridge/rounded tip	+	6/6 (100%)	7/14 (50%)
Prominent lower lip	−	4/6 (67%)	13/17 (76%)
Prognathia	+	0/4 (0%)	7/10 (70%)
Extremities			
Hands/feet abnormality	+	3/6 (50%)	15/18 (83%)
Other			
Gynecomastia	−	0/4 (0%)	5/12 (42%)
Hypogonadism/genital abnormalities	−	1/6 (17%)	14/17 (82%)

*CNS* central nervous system

In conclusion, we have identified a patient with a novel *CUL4B* splice site variant and demonstrate how *in silico* tools might be useful to initially assess intronic variants with unknown significance. We also suggest that patients with *CUL4B* variants might show less specific clinical features in early childhood, which slowly progress over time.

## Data Availability

The relevant data from this Data Report are hosted at the Human Genome Variation Database at 10.6084/m9.figshare.hgv.2618.
